# Supplementary material for: A UMLS-based spell checker for natural language processing in vaccine safety
Source: BMC Med Inform Decis Mak. 2007 Feb 12;7:3. doi: 10.1186/1472-6947-7-3 (PMC1805499; doi:10.1186/1472-6947-7-3)
Supplement: Additional file 13 — RAP application directory. Sets up directory for RAP (RDF application for PHP) source code [file 1472-6947-7-3-S13.gz › rap/doc/phpdoc/elementindex_rdql.html]

xml version="1.0" encoding="iso-8859-1"?


## [rdql] element index

### Package indexes

- model
- dataset
- infModel
- resModel
- ontModel
- syntax
- utility

All elements
  

c
g
h
m
n
p
q
r

c

top

countResults
:   RdqlResultIterator::countResults() in RdqlResultIterator.php

    Returns the number of results.

current
:   RdqlResultIterator::current() in RdqlResultIterator.php

    Returns the current result.

g

top

getCurrentPosition
:   RdqlResultIterator::getCurrentPosition() in RdqlResultIterator.php

    Returns the current position of the iterator.

getResultLabels
:   RdqlResultIterator::getResultLabels() in RdqlResultIterator.php

    Returns the labels of the result as array.

h

top

hasNext
:   RdqlResultIterator::hasNext() in RdqlResultIterator.php

    Returns TRUE if there are more results.

hasPrevious
:   RdqlResultIterator::hasPrevious() in RdqlResultIterator.php

    Returns TRUE if the first result has not been reached.

m

top

moveFirst
:   RdqlResultIterator::moveFirst() in RdqlResultIterator.php

    Moves the pointer to the first result.

moveLast
:   RdqlResultIterator::moveLast() in RdqlResultIterator.php

    Moves the pointer to the last result.

moveTo
:   RdqlResultIterator::moveTo() in RdqlResultIterator.php

    Moves the pointer to a specific result.

n

top

next
:   RdqlResultIterator::next() in RdqlResultIterator.php

    Returns the next result array.

p

top

parseQuery
:   RdqlParser::parseQuery() in RdqlParser.php

    Parse the given RDQL query string and return an array with query variables and constraints.

previous
:   RdqlResultIterator::previous() in RdqlResultIterator.php

    Returns the previous result.

q

top

queryModel
:   RdqlMemEngine::queryModel() in RdqlMemEngine.php

    Perform an RDQL Query on the given MemModel.

queryModel
:   RdqlDbEngine::queryModel() in RdqlDbEngine.php

    Perform an RDQL Query on the given DbModel.

r

top

RdqlDbEngine
:   RdqlDbEngine in RdqlDbEngine.php

    This class performs as RDQL query on a DbModel.

RdqlEngine
:   RdqlEngine in RdqlEngine.php

    Some general methods common for RdqlMemEngine and RdqlDbEngine

RdqlMemEngine
:   RdqlMemEngine in RdqlMemEngine.php

    This class performes as RDQL query on a MemModel.

RdqlParser
:   RdqlParser in RdqlParser.php

    This class contains methods for parsing an Rdql query string into PHP variables.

RdqlResultIterator
:   RdqlResultIterator in RdqlResultIterator.php

    Iterator for traversing Rdql results.

RdqlResultIterator
:   RdqlResultIterator::RdqlResultIterator() in RdqlResultIterator.php

    Constructor

RdqlDbEngine.php
:   RdqlDbEngine.php in RdqlDbEngine.php

RdqlEngine.php
:   RdqlEngine.php in RdqlEngine.php

RdqlMemEngine.php
:   RdqlMemEngine.php in RdqlMemEngine.php

RdqlParser.php
:   RdqlParser.php in RdqlParser.php

RdqlResultIterator.php
:   RdqlResultIterator.php in RdqlResultIterator.php

c
g
h
m
n
p
q
r
